# Barrier, weakness and utilization of pre-pregnancy clinic services

**DOI:** 10.1186/s13690-017-0236-2

**Published:** 2017-12-25

**Authors:** Mizanur Rahman, Natazcza Abdul Rahim, Mohd Taha Arif

**Affiliations:** Department of Community Medicine and Public Health, Faculty of Medicine and Health Sciences, Universiti Malaysia Sarawak, Kota Samarahan, Sarawak Malaysia

**Keywords:** Barriers, Perception, Pre-pregnancy care, Sarawak

## Abstract

**Background:**

Despite being one of the plausible measures towards achieving Sustainable Development Goals (SDGs), various issues pertaining to pre-pregnancy clinic (PPC) services still need to be pondered upon. Based on this view, an attempt was made to identify and understand the barriers and weaknesses of current utilisation of pre-pregnancy care services, since its establishment and implementation in Sarawak from the year 2011.

**Materials and methods:**

This cross-sectional study was conducted in selected health care facilities throughout Sarawak. A multistage cluster sampling technique was followed to select the health facilities. An unstructured open-ended questionnaire was administered as a part of quantitative data analysis. The open-ended questions were administered to get the in-depth perceived views and current practice of utilisation of pre-pregnancy clinic services. A total of 553 clients from nine selected health care facilities gave their feedback. The results of the study were narrated in textual form and a thematic analysis was done manually.

**Results:**

The identified themes for perceived barriers for utilisation of pre-pregnancy care were perception, attitude and acceptance of PPC services, socio-economic issues, services and client factors. The perceived weaknesses of the services are listed under two main themes: working environment and service factors, whereas, the strength of services produced three thematic areas which are preparation for pregnancy, prevention of mortality and morbidity and comprehensive services.

**Conclusions:**

Though there is ample evidence that pre-pregnancy services are beneficial for maternal health and wellbeing, various issues still need to be addressed for the improvement of the quality of services. Lack of awareness among clients, socio-economic barriers, lack of resources, organisational barriers and perceptions towards family planning issues are some of the issues which need to be addressed. Nonetheless, promotional and health educational activities are important keys; in ensuring the sustainability of the services.

## Background

Preconception care is aimed at identifying and modifying biomedical, behavioural and social risks through preventive and management interventions [1]. The major key components of preconception care are risk assessment and health promotion apart from the provision of medical and psychosocial interventions. Every woman should be prepared with their own reproductive life plan, based on their own values and resources [1].

Pre-conception care can be defined as “*interventions that optimize women’s health before pregnancy with the intent to improve maternal and newborn health outcomes” or “a set of interventions that aim to identify and modify biomedical, behavioural, and social risks to a woman’s health or pregnancy outcome through prevention and management*” [2]. The main purpose of implementing the pre-pregnancy clinic (PPC) services is to prevent pregnancies which are unplanned, too early or too close [3].

Though pre-pregnancy care services were first established nationwide in the year 2002, which was later expanded to Sarawak in the year 2011, the rate of utilisation and knowledge pertaining to pre-pregnancy care among women in Malaysia remains unclear [4]. Moreover, those not utilising the service were having at least one chronic medical illness, which complicate their pregnancies and deliveries [5]. Hence, various barriers to the provision of pre-pregnancy services need to be explored. The main objective of this study was to obtain clients’ views on barriers and weaknesses of utilisation of Pre-pregnancy Care services in Sarawak.

## Methods

### Study design and sample selection

This cross-sectional study was conducted at nine selected public health care facilities throughout Sarawak, whereby seven primary healthcare facilities and two hospitals were included in the study. The health care facilities were chosen according to their locality in Sarawak based on administrative zones in Sarawak.The anticipated proportion of clients who were satisfied with healthcare services were 86%. The calculated sample size was 183, which was then inflated by adding 20% attrition rate. Hence, the total minimal sample from each zone would be 200 samples. Finally, a total of 600 sample estimated getting the good estimate after multiplying by three. A total of 553 clients were selected by a multistage cluster sampling procedure using the client registries as the sampling frame. Clients who were Malaysians and consented to participate in the study were included as respondents, whereas clients of pre-pregnancy clinics with pre-existing mental illness (depression or anxiety) were excluded from the study.

### Sampling procedure

#### Selection of healthcare facilities (stage 1)

##### Selection of primary health care facilities

Fifteen primary healthcare facilities which were served by Family Medicine Specialists are available throughout Sarawak. A simple random sampling method (casting of lots) was used for selecting two primary health care facilities from each respective zone of Sarawak. Hence, a total of six primary health care facilities were included in this study.

##### Selection of hospitals

A total of 22 government hospitals are available throughout Sarawak. Three main reference hospitals for each respective zone (purposive sampling method); namely Sarawak General Hospital (South Zone), Sibu General Hospital (Middle Zone) and Miri Hospital (North Zone) were included in the study. Nonetheless, Miri General Hospital was replaced by KKIA Bandar Miri; as the service was only provided at the latter health care facility.

#### Selection of clients of pre-pregnancy clinic (stage 2)

##### Sampling procedure for clients of pre-pregnancy clinic

A systematic random sampling procedure is done for the clients of pre-pregnancy clinic by using the total number of clients in the registries as the sampling frame. A total of 200 samples would be collected from each zone whereby 50 samples would be collected from the hospital and 150 samples from primary health care facilities. Hence, a total of 600 pre-pregnancy clinic clients would be included for this study. Detailed sampling procedure illustrated in Table 1.

**Table 1:** Sampling procedure for selection of health care facilities, clients of pre-pregnancy care clinic

Stages of sampling process	Zones	Total of health care facilities for each zone		Total Samples		Sampling procedure
		Primary health care	Hospitals	Primary health care	Hospitals	
First stage Selection of health care facilities	South	7	8	2	1	Primary health care facilities Simple random sampling Hospitals Purposive sampling
	Middle	5	9	2	1	
	North	3	5	2	1	
	Total	15	22	6	3	
Second stage Selection of clients	Zones	Total targeted samples		Total samples		Simple systematic random Sampling
	South	150	50	200		
	Middle	150	50	200		
	North	150	50	200		
	Total	450	150	600		

### Data collection and analysis

An unstructured open-ended questionnaire was administered to get the in-depth perceived views and current uses of pre-pregnancy clinic services, which were based on a questionnaire used in a study entitled “Preconception care: Practice and beliefs of primary care workers” by Heyes et al. [6]. which was conducted at Barnsley Health Authority area, United Kingdom [6]. The questions are: a) What are the barriers that you face, while receiving the pre-pregnancy care services? b) What do you think are the weaknesses of pre-pregnancy care services? c) What do you think are the strengths of pre-pregnancy care services? A few questions were put to the respondents to further validate their written answers and to ensure that their answers did not deviate from the objectives of the questions. All the information was typed written into Microsoft Excel 2013. The data was coded by using thematic data coding method and the results of thematic analysis were presented in narrative form. The quantitative data on respondents’ socio-demographic characteristics was analysed by using IBM Statistics Software Version 22.0 and tabulated accordingly.

### Ethical consideration

The study proposal was approved by the Technical Review Committee of the Department of Community Medicine and Public Health, Universiti Malaysia of Sarawak (UNIMAS) and the Medical Research and Ethics Committee (MREC). Ethical clearance was also obtained from the Institutional Review Board (IRB) of the Faculty of Medicine and Health Sciences, UNIMAS and Institute of Public Health (IPH), Malaysia. All the participants have been informed about the study objectives and outcome. All were assured that no part of their name, address and identification number will be disclosed publicly. Data analysis was done anonymously. Informed written consent was obtained from the respondent before data collection.

## Results

### Socio-demographic characteristics of respondents

Table 2 showed that most of the respondents were Malays (39.4%), Muslims (46.7%) and the mean (SD) age of the respondents was 30.6 (7.2) years. A few of the respondents were adolescents (15.4%). Almost all of them (97.8%) had at least primary education, though over half (55.7%) of them were housewives. Almost all of the clients were married (95.5%) and a small fraction of them were either single (0.7%) or living with partners (2.7%) or divorced (1.1%). A total of 30 clients (5.4%) were grand multiparas, whereas 9.9% (*n* = 55) were nulliparous. Nonetheless, over half (55%) had ever planned their pregnancies before.

**Table 2:** Socio-demographic characteristics of the respondents

Characteristics	n	%
Mean (SD) Age in years	30.57 (7.2) years	Min, 16; Max 49
Ethnicity		
Malay	218	39.4
Non-Malays	335	60.4
Religion		
Islam	258	46.7
Christianity	255	46.1
Others	40	7.2
Level of education		
No education	12	2.2
Primary	56	10.1
Secondary	316	57.1
Higher education (STPM/Diploma/Degree)	169	30.6
Working status		
Employed	240	43.4
Unemployed	308	55.7
Student	5	0.9
Marital status		
Single/Unmarried	4	.7
With partner/Unmarried	15	2.7
Divorced	6	1.1
Married	528	95.5
Mean (SD) Number of Pregnancy	2.42 (1.520)	Min = 0, Max = 10
Ever planned for pregnancy		
No	304	55.0
Yes	249	45.0

### Barriers and weaknesses of pre-pregnancy care services

The main themes and subthemes for barriers, weakness of utilisation of pre-pregnancy care services are as listed in Table 3 below.

**Table 3:** Main themes and subthemes for barriers and weaknesses of pre-pregnancy care services

Main theme	Subtheme
Clients Factors	• Unaware of the services
	• Family Commitments
	• Do not understand health care providers
	• Accessibility to nearest health care facility
	• Unwilling to plan for pregnancy
	• Unplanned pregnancy
	• Patient attending clinic not for PPC
	• Patient aware, but refuse to utilise service
	• Distance from home
	• Accessibility to nearest facility
	• Socio-economic status
	• Women’s perception
	• Husband’s acceptance
	• Religiously not permitted
	• Time constraint
Services factors	• Lack of facilities at rural clinics
	• Health care attitudes towards clients
	• Lack of discussion between health care providers and clients
	• Unconducive environment
	• Untrained staff
	• Lack of resources
	• Lack of privacy
	• Longer stay in clinic
	• Lack of collaboration
	• Disorganised system

### Clients’ factors

A lot of women are unaware of pre-pregnancy services despite its establishment over the last five years. Lack of promotional activities and non-corporation of the service providers, commitment to the other health programs were few of the reasons stated by the respondents.

“*…. I am unaware at first of the existence of the services, as the services were not promoted. Never seen any posters or educational materials on it. I got to know the services by a friend of mine, who is a client of PPC.”*

*(Housewife, 28).*

*“..... I am unaware at first that the consultations provided by the medical officers are part of the services; as I was not properly explained on the services. Besides, the services are incorporated with my routine follow-up; for thyroid function monitoring”.*

*(Housewife, 27).*

Though clients may be keen with the services, family commitments were hindering them from utilizing the service. Furthermore, they may be attending the clinic for various other reasons.

*“…...I am aware and interested in it…but (sigh)...I am too preoccupied with the kids and household chores. Besides, I usually go to the clinic because of the kids…bringing them for follow ups….so…no opportunity to get my PPC services.”*

*(Housewife, 32).*

Factors such as language barriers, clients’ academic qualifications, contents of the services and lack of knowledge on PPC services among the staffs themselves are affecting the dissemination of information to the clients.

*“… Cannot really understand the content of the services…my Bahasa is not that fluent. Better to provide the pamphlets in Iban language….some of the terms are jargons to me.”*

*(Housewife, 22).*

Accessibility to the nearest health care facility is the key factor which will determine the utilisation of any health care services. However, factors such as financial constraint, transportation, poor infrastructure and distance from healthcare facility remained as important barriers to health care services.

*“….I had to come to Kuching, just to attend my pre-pregnancy care follow-up; whereas I am from Sri Aman. Some of the services can only be found at Sarawak General Hospital. That requires expenses...I do not have my own earnings and my husband does not earn that much. Furthermore, it is quite a distance from our home…and we do not have our own transportation.”*

*(Housewife, 23).*

The unwillingness of women to plan for pregnancy and unplanned pregnancy were the most integral issues which need to be addressed. Moreover, lack of perception of risks among young, healthy women provides another challenge to the services. Some of them had the perception that the services are only meant for infertile women or those with medical problems. Others perceived that concerns on health issues and lifestyles should only arise after getting pregnant.

*“…. Initially, I was not interested in the services. Thought that I am not at risk, as I am still young and I still want more kids. Moreover, I always thought that the services are for infertile couples and for those who have medical problems. Never thought about health, till I was pregnant last year.”*

*(Self-employed, 25).*

Teenage mothers were a bit apprehensive to use the services due to stigmatisation towards them. Furthermore, most of the healthcare providers were unskilled in dealing with adolescents and were judgmental towards them. Some of the respondents had the perception that the services should meet their individual needs apart from being provided at the right timing and suitable platform. Nonetheless, previously unplanned pregnancies also hindered them from using the services.

*“…..I am a bit afraid to attend the clinic. I had a bad experience during my first antenatal clinic follow-up…the nurses may have bad impressions towards me.”*

*(Housewife, 16).*

*“…Though I agree to be registered with the services, I think it is too soon for me to think of having another kid. Furthermore, my kid is barely a month old. I might get the services later; when my kid is slightly older.”*

*(Housewife, 23).*

*“…I never knew that consultation on environmental health issues is included…need the services as I am working at a factory.”*

*(Factory worker, 25).*

*“….I was advised to use the services, two years ago as I planned to get pregnant. But then, I conceived earlier! So…I decided to register earlier this time…do not want to experience the complications that I had during last pregnancy.”*

*(Housewife, 36).*

Few of clients’ spouses were unaccepting towards the client’s decision to utilize the pre-pregnancy services. Family planning is regarded as women’s responsibility as they have more options and access to the services. Nonetheless, there was also the lack of discussions between women and health care providers pertaining to pre-pregnancy care issues.

*“….My husband did not agree with my decision to utilize the services. He claimed that the services are a waste of time….and the waste of money as well. He is also busy at workplace…besides, he thinks that he does not have to be there, listening to women's issues. He also said that I should be the one practicing family planning…as there are a lot of services available for women and I am just staying at home…free most of the time.”*

*(Housewife, 28).*

*“….Most women are shy to talk about their intention to get pregnant…including myself. Initially, I was dumbfounded when I was asked whether I am planning to get another kid. How to discuss on this issue when I myself were not even thinking of family planning?”*

*(Housewife, 27).*

Religious beliefs also cause a significant barrier for pre-pregnancy services (15).

*“….Though it is not prohibited to plan for pregnancies, it is hard to make a decision on ‘legal abortion’; especially when it is going to affect the health of the mother.”*

*(Government servant, 27).*

### Services factors

Most of the clinics were not operating on a daily basis though the referrals of clients can be done every day. This cause inconvenience to working women as they were only available after office hours or during the weekend.

*“…Difficult for me to attend my follow ups, as I am busy with work. Need to take the time off, or apply for leave.”*

*(Government servant, 35).*

The clients are also sceptical of the program, as most of the health care providers are untrained and had the lack of knowledge on pre-pregnancy care. Nonetheless, the clients did acknowledge that there was lack of resources for the programs; namely staff and stocks of contraceptive methods.

*“…Pity the nurse…all alone handling the program. She also needs to attend to other matters. Moreover, she is untrained…unable to clarify most of the clients’ questions. The stocks for OCPs are also limited….once, she actually had to owe her clients, their pills.”*

*(Private sector worker, 33).*

Moreover, other factors such as long stay in the clinic, time constraint, unsuitable consultation areas and lack of privacy also discouraged them from utilizing the service.

*“…I would love to stay longer in the clinic, but I need to return back home by noon, or else I do not have any transportation back home. I need to catch the van…”.*

*(Housewife, 20).*

*“... I need to spend a couple more hours at the clinic if it happens that I need to attend my PPC follow up. Quite tiring for me….”*

*(Housewife, 35).*

*“…The patients are counselled in the treatment room…so hot and stuffy. The room is not suitable at all.”*

*(Housewife, 30).*

*“…The nurse had to share rooms with others…lack of privacy for clients. Other patients can hear what the providers are telling you; besides…health information should be confidential; should not be handled by a lot of staffs.”*

*(Housewife, 34).*

The clients also claimed that there is lack of collaboration between various health departments; and different level of health care facilities. The delivery system for the services was also disorganised.

*“…. Never been referred by medical department, for PPC services before. I got to know the program while attending my regular follow-ups at the primary health care clinic.”*

*(Lecturer, 30).*

*“….It is such a hassle that the clients need to go to other room for pre-pregnancy care services…the appointment system also needs to be improved…better to go through a counter. The stamping method is also ineffective, the staff themselves were unaware of my appointment. A lot of clients default their follow ups due to this problem.”*

*(Government servant, 28).*

*“…I had requested to be discharged to a clinic nearer to my house. Apparently, it was not done earlier; as the staff were unaware that the services are available at that clinic”.*

*(Housewife, 38).*

*“…As a first timer….I may get lost in this clinic. I do not even know where the room is! No signage on the door! So…in the end….I defaulted the follow-up.”*

*(Housewife, 28).*

Nevertheless, few of the respondents did voice the need to have a delegated centre or facility either within the compound of the clinic or at a separate locality.

*“…there is no dedicated room or building within the compound of the clinic…it should be more private and provide comfort for the clients. A specialized center at a separate location would be nice.”*

*(Housewife, 24).*

### Strength of pre-pregnancy care services

The generated main themes and subthemes for a strength of pre-pregnancy care services are as listed in Table 4 below:

**Table 4:** Main themes and subthemes for strength of pre-pregnancy care services in Sarawak

Main Themes	Subthemes
Preparation for pregnancy	• Optimise health of women before pregnancy • Mental preparation for pregnancy • Early planning for antenatal care/delivery • Help for infertile couple
Prevention of mortality and morbidity	• Early detection of health risks/medical problems • Monitoring health of women • Contraception
Comprehensive services	• Incorporation of other services • Health educational/promotional tool • Cover all age group • Friendly and experienced staffs

### Preparation for pregnancy and prevention of mortality and morbidity

Pre-pregnancy services are perceived as an aid to reduce the incidence of maternal and child mortality. It helps to identify health risks, optimise women’s health prior to pregnancy and act as an aid for infertile couples. Moreover, early provision of contraception can be given to those who are unfit for pregnancy. It provides for future parents to get mentally prepared for pregnancy, delivery and child care. Few respondents did agree that they should have been promoted with the services; much earlier.

*“…My diabetic control can be optimized, prior to my pregnancy. After all, the medication needs to be changed if I get pregnant…the program allows me to do just that. At least, the doctor can plan on how I should be monitored throughout my pregnancy, delivery and post-natal period. He or she can offer contraception; if needed.”*

*(Government servant, 38).*

*“…The services helped me to reduce the risk of pregnancy and delivery towards my health….I delivered my child prematurely due to pregnancy induced hypertension last year…hence, I want to make sure that my baby will be delivered safely this time…can help me to get mentally prepared for this pregnancy…Just wished that I had known the services earlier on.”*

*(Government servant, 30).*

*“…I was interested in the services, as I had problem conceiving; after my last childbirth. Hope that I can get pregnant after this…”.*

*(Housewife, 36).*

### Comprehensive services

Furthermore, other health care services are incorporated with the service hence enabling the clients to attend their other routine follow-ups apart from reducing the defaulters’ rate.

*“…incorporation of other health services with pre-pregnancy care services; enable me to get other services; while attending my PPC follow up. So...women will not default their appointment.”*

*(Private sector worker, 34).*

### Health promotional tool

Pre-pregnancy care was also seen as a health educational and promotional tool, among the public. Nonetheless, friendly and knowledgeable staff and coverage of all age group of the public were few of the plausible aspects of the service.

*“….I have learnt a lot on various health issues, such as family planning, high-risk behaviour and healthy lifestyle. Both male and females from all age groups can get the benefits of the program. The staff are also friendly and experienced.”*

*(Government servant, 23).*

## Discussion

### Strengths of pre-pregnancy care services

Similar to a previous study by Mazza et al. [7], pre-pregnancy care service was regarded as a tool to optimise women’s and child’s health status which subsequently reduce both maternal and child’s mortality and morbidity rates. Pre-pregnancy care was also perceived as a health educational and promotional tool for both healthcare providers and clients which was also described by Mazza et al. [7]*.* The findings of this study also corresponded with the findings by Mazza et al. [7], whereby men’s involvement in the service should be emphasised on in view of higher prevalence of high risk behaviour among them, as what was supported by a housewife:

*“.....My husband should get involved (in pre pregnancy care services). It may help my husband to quit his smoking habit.”*

*(Housewife, 23).*

The providers were perceived as friendly and experienced, which was supported by the findings by Abu Talib et al. [8]. This may be attributed by the fact that the nurses who were attending them particularly at the Maternal and Child Health clinics were trained in provision of family planning services.

### Barriers and perceptions on pre-pregnancy care services

Accessibility to the nearest health care facilities and socio-economic barriers still remain as the most important barrier to health care services [9–17] around the globe. Long waiting time and various cultural barriers [9, 11, 15–17] were also mentioned in this study. Though the prevalence of usage of contraception (19.9%) among women are still considered quite low [5], awareness among them are on the rise.

The perception that the services were not meant to cover all age group should be corrected, as adolescents should be included as a targeted group, and should not be neglected in provision of pre-conception care. This will subsequently lead to lack of involvement among adolescent girls as what was perceived in a study in Iran [18]. Adolescents’ involvements in pre-pregnancy care services are seen to be an integral measure; towards reducing the rate of teenage pregnancies [18]. In this study, younger respondents (10.4%) may disagree with the importance of preconception care; which may be attributable to lack of knowledge and lesser encounter with health care facilities [19].

Unplanned pregnancies, women’s limited knowledge on pre-pregnancy care issues and women’s behaviour of seeking for health care only after being pregnant were among the most perceived barriers of pre-pregnancy care services among women in Sarawak which were also demonstrated in previous studies. A recent study by Kasim et al. [19] among women in Kelantan revealed that only half of the respondents had good knowledge on pre-pregnancy issues. Various other studies [20, 21] also did support lack of knowledge among women and providers. Pre-pregnancy care services was perceived to be more useful for infertile couples or those who were planning to conceive which was demonstrated in various previous studies [22–24].

Few of high risk women in this study were keen for a pregnancy despite of knowing the possible risks. This was further supported by a study among diabetic women, which stated that their desire for a pregnancy as one of the reasons for not utilising pre-pregnancy care or continuation of contraception [25]. Surprisingly, high risk women in this study would rather practice contraception relative to attending pre-conception counselling sessions. Nonetheless, contraception was provided to women without further understanding on basic issues of reproductive systems, its anatomy and physiology, and the concept of preconception care. Subsequently, pre-conception issues and its services were not being emphasised on and was not seen as a family planning method by most women [18].

*“….Initially, I did not know what was PPC; until I was introduced with it last year. During the last pregnancy, I was automatically offered with pills and injections as contraception. Decided to use it (PPC) this time.”*

*(Housewife, 28).*

Additionally, clients were also sceptical with the outcomes and effectiveness of the program.

*“…..Unsure whether I can get pregnant after this two years (PPC)...would like to get pregnant as soon as possible.”*

*(Housewife, 24).*

Provision of pre-pregnancy care is both time and resource consuming, due to a considerable amount issues which need to be addressed alongside with other primary health care services which need to be provided for the clients [20]. Longer stay in the clinic and the need of more visits to the healthcare facilities was unappealing to women, which subsequently cause lack of women’s commitment on the services; as implied by a private sector worker.

*“Did not see the point of attending the clinic...most of the time, the nurses were just filing up the forms; apart from providing me with folic acids...”*

*(Private sector worker, 29).*

Low service uptake eventually lead to lack of confidence and skills among the providers as it hindered them from developing routines and gaining experiences in provision of pre-pregnancy care services [20]. Subsequently, they were perceived by the clients as being incompetent or was performing badly in provision of the service; which lowered the perception on the quality of service. Few of important aspects of preconception care such as exposure to occupational health hazards and environmental hazards and genetic counselling were found to be neglected in provision of the service. It was apparent that few women were aware of these potential hazards and its adverse outcomes.

*“...Wanted to know more on the possible effects of chemicals to me and pregnancy..as i am mostly dealing with chemicals at workplace..erm...but the doctors and nurses seemed to be not well versed with the topic....”*

*(Factory worker, 29).*

Lack of awareness among women pertaining to pre-pregnancy issues is attributable to lack of promotional and health educational activities [4]. Women was claimed to have lack of initiative to search for relevant information on pre conception issues and their main concerns were only on getting pregnant or their fear on failure of conception. However, this scenario was attributed by women’s lack of knowledge on pre-pregnancy care issues, as what was claimed by a private sector worker.

*“....I did not know what to ask the providers or where to search for relevant information on the service; as I had limited knowledge...even if I have the internet, I will not know what to Google.”*

*(Private sector worker, 29).*

The findings of this study contradict the findings by Mazza et al. [22], whereby the clients were uncomfortable with opportunistic counselling; as their intention for pregnancy was being asked in an abrupt manner.

“.....I was suprised when my nurse asked me whether I was planning to get pregnant...which was too abrupt to me....I could not give the answer then...felt uncomfortable...I do not usually share this with other people..especially as it was done unprivately.”

(Housewife, 30).

Women’s perception on pre-pregnancy issues should be addressed as it act as an important barrier towards the services [22–24]. Women’s perception on pregnancy as a natural event, which requires no preparation by the involved couple; become an important barrier of pre-conception care. Women tend to keep their intent of getting pregnant to themselves which act as a defence mechanism against disappointment and avoidance of false expectations on them and their spouses. Moreover, providers had lack of proactivity and motivation in term of providing pre-conception services. Services were offered only once it was inquired by women, rather than being provided spontaneously by the providers. Hence, women had the impression that information on preconception issues were not readily available and they had to become proactive to gain access to that information. Hence, health educational and promotional activities should be emphasised on as positive attitudes toward health seeking behaviour were demonstrated, once essential and precise information related to pre-conception issues were disseminated among women. Nonetheless, most women do have positive attitudes toward pre-pregnancy services though they are reluctant to utilise the services [6]. Discussion on family planning with women should be done at the right timing and by using the suitable platform. In addition, the services should be provided according to women’s individual needs and their socio-economic status [23, 24].

Unlike in other studies, most of the clients were of middle aged group (over 40 years old) which were of the older target group of clients. This may be attributed by the fact that more women of middle age group were recruited as clients compared to younger, healthier women. This may support the findings by Ojukwu et al. [21], whereby older women of higher educational level and socioeconomic status had better knowledge on preconception health and were more health conscious hence were more likely would seek preconception care from their providers. In contrast, students were reported as being more educated on preconception care issues therefore, will be less likely to seek for advice from health providers.

Men’s negative perceptions on pre-pregnancy care services also act as an important barrier of its services in Sarawak. As supported by previous studies [26–29], lack of emphasis on men’s health issues and perception that family planning is solely the responsibility of women were also present in this study. Furthermore, women were perceived as having more opportunities to attend the counselling sessions apart from having more options for contraceptive methods. Provision of specific preconception care to men was rare, except in few contexts such as for counselling or screening for hereditary conditions. More evidence-based information was needed to show the effectiveness of preconception interventions for men before this became more of a focus within consultations.

This study differed from the study by Abu Talib et al. [4], which stated that daily commitments were not a barrier for utilising the services. In that particular study, the respondents did receive supports from their family members for provision of care for their children and completion of household chores. Furthermore, the long waiting time were seen as an opportunity to return back home in order to complete their daily tasks [11]. These scenarios were rare in Sarawak whereby distance from the healthcare facilities, lack of infrastructures and transportation issues should be taken into consideration. Nonetheless, there was a rising concern that this service may be perceived by the public as a ‘nanny state’ as an effort to instil positive behaviours towards family planning and high risk behaviours, as supported by a private sector officer.

*“....It felt as if we were forced or being guided to practice any form of contraception or family planning methods.”*

*(Private sector worker, 32).*

The delivery systems of pre-pregnancy care were disorganised and inefficient. It was shown that the referral system was not being coordinated accordingly between the pre-pregnancy care providers with the primary care providers and various other health departments resulting in lack of referrals or late referrals. This will subsequently cause suboptimal and inefficient delivery of healthcare services. Moreover, most consultations at primary health care settings were mostly restricted to 10 min appointments, which were inadequate for provision for both pre-conception care and other consultations [21].

Unscreened access to their medical records did arise concerns among the respondents with sensitive issues and it was affecting the uptake of pre-pregnancy services. Assurance of confidentiality and privacy remained as important issue, as most of the clinic settings were unconducive; especially for the older healthcare facilities. A more conducive consultation room should be available in ensuring that the consultation session can be done privately and in comfort.

Though nurses and medical officers were the important providers of pre-pregnancy care services, medical officers were perceived as the more important providers by the respondents; which correspond to a study by Tokunbo et al. [30]. Absence of medical officers in the pre-pregnancy consultation rooms, caused lack of assurance among the clients on the efficacy of the service as most of the health issues still need to be referred to the medical officers. Yet, the services which were provided by the medical officers were rarely perceived as part of pre-pregnancy care services. This may be due to the incorporation of the services with other healthcare services. More efforts should be done by the staff of the outpatient department, in identifying high-risk women. An updated census on high-risk women should be made available and shared with the OPD units in ensuring that all high-risk group women are either on contraception or being registered under this program.

Untrained staff, poor delegation of staff and lack of confidence among staff were among the factors which affect the quality and trust of the public towards the services. [11, 16, 31]. Hence, more training and resources should be allocated for the program in ensuring that the services can be accessible at most health care facilities in Sarawak. Other factors such as transportation, infrastructural issues, long waiting time, religious and cultural factors, lack of facilities, daily commitments, confidentiality issues and previous bad experiences were also reported in this study [11–15, 31–35].

Husband’s involvement in the services should also be improved [7]. Various measures such as inclusion of male health issues, flexible schedule, daily consultations and provision of the services at Klinik 1Malaysia should be considered. A registration counter needs to be established and a dedicated medical officer should be in charge of the program. A more systematic delivery system should be implemented and guidelines for referrals should be made available.

### Limitation of study

Few limitations were encountered during this study. Firstly, the collection of data was done based on a face to face interview which might lead to social desirability bias. Recall bias may be present in this study, as the respondents need to recall few of the required information. Additionally, the respondents may refuse or did not complete the open ended section which may lead to incomplete collection of data.

The findings of this research would not be able to be applied to the whole population of pre-pregnancy care users and health care providers in Sarawak, as healthcare personnel from other departments of public hospitals such as Medical and Surgical Department, private healthcare services and other public or private agencies were excluded in this study. Selection of the healthcare personnel were also based on ‘convenience sampling’ method causing disproportionate involvement of the healthcare providers. Furthermore, not all of them were available on the day of field visit. There was also lack of involvement of young and healthy women in this study, who may provide different perceptions pertaining to the service relative to those with underlying medical illness or previous bad obstetrics outcomes.

The involved healthcare facilities were of mainly Type I and Type II clinics in Sarawak with easy accessibility and well equipped. Hence, common barriers of healthcare services were less likely to be perceived by both clients and providers. More remote healthcare facilities should also be included in this study; which may provide a different perspective on the barriers of healthcare.

## Conclusion

Though there is ample evidence that pre-pregnancy services are beneficial for maternal health and wellbeing, various issues still need to be addressed for the improvement of the quality of services. Lack of awareness among clients, socio-economic barriers, lack of resources, organisational barriers and perceptions towards family planning issues are some of the issues which need to be addressed. Nonetheless, promotional and health educational activities are important keys; in ensuring the sustainability of the services.

## Abbreviations

FMHS: Faculty of Medicine and Health Sciences
IPH: Institute of Public Health
OPD: Out Patient Department
*PPC*: Pre-pregnancy Care
SDGs: Sustainable Development Goals
UNIMS: Universiti Malaysia Sarawak

## References

[1] Lu M (2007). Recommendations for preconception care. Am Fam Physician.

[2] Centre for Disease Control and Prevention. Preventing and Managing Chronic Disease to Improve the Health of Women and Infants [Fact sheet]. 2006. Retrieved from http://www.idph.state.ia.us/hpcdp/common/pdf/family_health/2012_cdc_factsheet.pdf. Accessed 2 Jan 2015.

[3] World Health Organization. Preconception care: Maximising the gains for maternal and child health. 2013. Retrieved from the World Wide Web: http://www.who.int/maternal_child_adolescent/documents/preconception_care_policy_brief.pdf. ​Accessed 6 Jan 2015.

[4] Abu Talib R, Idris IB, Sutan R, Ahmad N, Bakar NA (2016). Exploring the determinant of pre-pregnancy care services usage, reproductive ages women in Kedah, Malaysia. Int J Public Health Res.

[5] Muhammad NM, Ruziaton H, Nuraini D, Izan HI, Norizzati BIB, Mohamad RI, Mimi O (2014). Risk factors for women attending pre-pregnancy screening in selected clinics in Selangor. Malays Family Physician.

[6] Heyes T, Long S, Mathers N. Preconception care: practice and beliefs of primary care workers. Fam Pract. 2004;21(1):22–7. 10.1093/fampra/cmh106 14760039

[7] Mazza D, Chapman A, Michie S. Barriers to the implementation of preconception care guidelines as perceived by general practitioners: a qualitative study. BMC Health Serv Res. 2013;13(1):36. Retrieved from 10.1186/1472-6963-13-36. Accessed on January 8, 2015 10.1186/1472-6963-13-36 PMC3565953 23368720

[8] Talib RA. Exploring the determinant of pre-pregnancy care services usage among reproductive ages women in Kedah, Malaysia. Int J Pub Health Res. 2016;6(2);719–26.

[9] Rosliza AM, Majdah M (2010). Male participation and sharing of responsibility in strengthening family planning activities in Malaysia. Malays J Pub Health Med.

[10] Singh, D., Lample, M., & Earnest, J. (2014). The involvement of men in maternal health care: cross-sectional, pilot case studies from Maligita and Kibibi, Uganda. *Reproductive Health*, *11*(1), 68. Retrieved February 8, 2015 from the world wide web doi:10.1186/1742-4755-11-68. 10.1186/1742-4755-11-68 PMC4167520 25192714

[11] Kronfol NM. Access and barriers to health care delivery in Arab countries: a review. East Mediterr Health J. 2012;18(12):1239–46. 10.26719/2012.18.12.1239 23301399

[12] Olayinka OA, Achi OT, Amos AO, Chiedu EM. Awareness and barriers to utilization of maternal health care services among reproductive women in Amassoma community, Bayelsa State. Int J Nurs Midwifery. 2014;6(1):10–15. doi:10.5897/ijnm2013.0108. Accessed 10 Feb 2015.

[13] Bakeera SK, Wamala SP, Galea S, State A, Peterson S, Pariyo GW. Community perceptions and factors influencing utilisation of health services in Uganda. Int J Equity Health. 2009;8(25. Retrieved from doi: 10.1186/1475-9276-8-25) Accessed on February 17 2015 10.1186/1475-9276-8-25 PMC2717964 19602244

[14] Otiniano A. D., Muthengi, E., Wakeel, F., Doan, L. C., Ramos D. E. (2006). Perceived Barriers to Preconception Care: Findings from Los Angeles Mommy and Baby (LAMB) Survey. Retrieved from http://publichealth.lacounty.gov/mch/lamb/Results/2007Results/APHABarrierstoPreconception%20Care_101208.pdf. Accessed 3 Jan 2015.

[15] Jack BW, Culpepper L (1991). Preconception care. J Family Pract.

[16] Okigbo C. Provider education: key to improving young women’s use of reproductive health services in urban Nigeria. Population Reference Bureau (PRB). Policy Fellows Working Papers Series; 2014. Retrieved from http://www.prb.org/pdf14/provider-education-in-nigeria.pdf. Accessed 30 Oct 2016.

[17] Bronstein, J. M., Felix, H. C., Bursac, Z., Stewart, M. K., Foushee, H. R., & Klapow, J. (2012). Providing general and preconception health care to low-income women in family planning settings: perception of providers and clients. Maternal and Child*.* 10.1007/s10995-011-0744-6 21258961

[18] Bayrami R, Roudsari R.L, Hamid Allahverdipour H, Mojgan Javadnoori M, Habibollah Esmaily H. Experiences of women regarding gaps in preconception care services in the Iranian reproductive health care system: a qualitative study. Electron Physician. 2016;8(11):3279–88. 10.19082/3279 PMC5217821 28070262

[19] Kasim R, Draman N, Kadir AA. Knowledge , Attitudes and Practice of Preconception Care among Women Attending Maternal Health Clinic in Kelantan. 2016;8(4):57–68.

[20] M’hamdi, H. I., van Voorst, S. F., Pinxten, W., Hilhorst, M. T., & Steegers, E. A. P. (2016). Barriers in the uptake and delivery of preconception care: exploring the views of care providers. Matern Child Health J, 21(1), 1–8. Retrieved from doi:10.1007/s10995-016-2089-7 10.1007/s10995-016-2089-7 PMC5226984 27423236

[21] Ojukwu O, Patel D, Stephenson J, Howden B, Shawe J (2016). General practitioners’ knowledge, attitudes and views of providing preconception care: a qualitative investigation. Ups J Med Sci.

[22] Mazza D, Chapman A (2010). Improving the uptake of preconception care and periconceptional folate supplementation: what do women think?. BMC Public Health.

[23] Tuomainen H, Cross-Bardell L, Bhoday M, Qureshi N, Kai J (2013). Opportunities and challenges for enhancing preconception health in primary care: qualitative study with women from ethnically diverse communities. BMJ Open.

[24] Van der Zee B, de Beaufort ID, Steegers EAP, Denktaş S (2013). Perceptions of preconception counselling among women planning a pregnancy: a qualitative study. Fam Pract.

[25] Rosliza AM, Majdah M (2010). Male participation and sharing of responsibility in strengthening family planning activities in Malaysia. Malays J Public Health Med.

[26] Singh, D., Lample, M., & Earnest, J. (2014). The involvement of men in maternal health care: cross-sectional, pilot case studies from Maligita and Kibibi, Uganda. *Reproductive Health*, *11*(1), 68. Retrieved February 8, 2015 from the World Wide Web doi:10.1186/1742-4755-11-68 10.1186/1742-4755-11-68 PMC4167520 25192714

[27] Narang, H., & Singhal, S. (2013). Men as partners in maternal health: an analysis of male awareness and attitude. *International Journal of Reproduction, Contraception, Obstetrics and Gynecology*, *2*(3), 388. Retrieved January 4, 2015 from the World Wide Web doi: 10.5455/2320-1770.ijrcog20130925

[28] Kabagenyi A, Jennings L, Reid A, Nalwadda G, Ntozi J, Atuyambe L. Barriers to male involvement in contraceptive uptake and reproductive health services: a qualitative study of men and women’s perceptions in two rural districts in Uganda. Reprod Health. 2014;11(1):21. Retrieved from doi: 10.1186/1742-4755-11-21. Accessed on January 10, 2015 10.1186/1742-4755-11-21 PMC3946591 24597502

[29] Murphy P, Phillips G, Hall A, Brooks S. Women’s Health Stats and Facts. [Fact sheet]. 2011. Retrieved from http://www.acog.org//media/NewsRoom/MediaKit.pdf.

[30] Tokunbo O, Abimbola O, Polite I, Gbemiga O (2016). Awareness and perception of preconception care among health workers in Ahmadu Bello University teaching university, Zaria. Trop J Obstet Gynaecol.

[31] Dehne KL, Riedner G (2001). Sexually transmitted infections among adolescents: the need for adequate health services. Reprod Health Matters.

[32] Ghafari M, Shamsuddin K, Amiri M (2014). Barriers to utilization of health services: perception of postsecondary school Malaysian urban youth. Int J Prev Med.

[33] Temel S, Birnie E, Sonneveld HM, Voorham AJJ, Bonsel GJ, Steegers EAP, Denktaş S (2013). Determinants of the intention of preconception care use: lessons from a multi-ethnic urban population in the Netherlands. Int J Pub Health.

[34] Chiang C, Labeeb SA, Higuchi M, Mohamed AG, Aoyama A (2013). Barriers to the use of basic health services among women in rural southern Egypt (upper Egypt). Nagoya J Med Sci.

[35] Scheppers E, van Dongen E, Dekker J, Geertzen J, Dekker J (2006). Potential barriers to the use of health services among ethnic minorities: a review. Fam Pract.

